# Sphingosine kinase 2 and multiple myeloma

**DOI:** 10.18632/oncotarget.17420

**Published:** 2017-04-25

**Authors:** Nigel J. Pyne, Susan Pyne

**Affiliations:** Strathclyde Institute of Pharmacy and Biomedical Sciences, University of Strathclyde, Glasgow, Scotland, UK

**Keywords:** sphingosine 1-phosphate, endoplasmic reticulum stress, unfolded protein response, autophagy, apoptosis

The survival of patients with Multiple Myeloma (MM) has improved due to the use of chemotherapeutic agents with autologous hematopoietic stem cell transplantation. However, the disease is incurable and relapse may occur within months. In addition, chemo-resistance to agents, such as bortezomib can occur and therefore there is an unmet medical need. Sphingosine kinase 2 (SK2), the enzyme that catalyses formation of the bioactive lipid, sphingosine 1-phosphate (S1P) has recently been identified as a viable target for therapeutic intervention in MM [1]. Indeed, the SK2 inhibitor, ABC294640, has been shown to induce the down-regulation of Mcl-1 and c-Myc leading to caspase-3-dependent apoptosis of MM cells [1]. Moreover, SK2 expression levels are increased in bone marrow CD138^+^ myeloma cells from patients [1].

Wallington-Beddoe et al. [2] in this issue of Oncotarget have analysed a larger gene expression data set from CD138^+^ bone marrow plasma cells from newly diagnosed myeloma patients compared to normal healthy controls. The authors found that there was increased expression of several genes involved in sphingolipid biosynthesis, including serine palmitoyltransferase, 3-keto-dihydrosphingosine reductase, alkaline ceramidase and SK2. The expression levels of a second sphingosine kinase isoform, SK1 were not changed. These findings suggest that SK2 is the predominant functional SK isoform in MM, and acts to remove ceramide to limit a ‘ceramide burden’ and to promote MM cell survival via S1P-dependent mechanisms. The latter might include SK2/S1P-regulation of nuclear histone deacetylase-1/2 (HDAC-1/2) [3] and telomerase reverse transcriptase (TERT) that sustains a replicative immortality programme in cancer cells [4]. Wallington-Beddoe et al. [2] have also made a very significant advance by demonstrating that SK2 inhibitors induce a caspase-3-dependent apoptosis of MM cells via an endoplasmic reticulum stress (ER stress)/sustained unfolded protein response (UPR). Moreover, they provide evidence that non-lethal doses of SK2 inhibitors (e.g. K145) and bortezomib can lead to synergistic sustained UPR and CCAAT/enhancer binding protein (C/EBP) homologous protein (CHOP)-induced apoptosis of MM cells. These findings are very important because they provide mechanistic evidence for the action of SK2 inhibitors in cancer cells. Moreover, they suggest that combined treatment with SK2 inhibitors and bortezomib might allow use of lower doses of each, thereby avoiding/delaying chemo-resistance and increasing the effectiveness of therapeutic intervention in MM.

Bortezomib promotes the accumulation of protein which overwhelms the folding capacity of the ER to initiate ER stress and sustain UPR and which subsequently leads to apoptosis of CD138^+^ myeloma cells. This involves dissociation of the ER chaperone binding immunoglobulin protein (BiP) from the lumenal domains of ER stress sensors, which are protein kinase R-like ER kinase (PERK), inositol-requiring kinase 1 (IRE1α) and activating transcription factor 6 (ATF6). Prolonged ER stress leads to sustained UPR activation that results in an increase in the expression of the pro-apoptotic transcription factor CHOP. Indeed, using a variety of MM cell lines, Wallington-Beddoe et al. [2] demonstrate that bortezomib induces sustained UPR as evidenced by increased BiP, XBP1s, p-eIF2α and CHOP levels. In addition, they demonstrate that the SK2 inhibitor, K145, induces ER stress, which is shown by increased expression of XBP1s and p-eIF2α. However, BiP is only modestly increased in response to K145 and this provides the molecular basis for the synergism with bortezomib by allowing complementarity in the activation of ER stress sensors that result in CHOP-dependent apoptosis. This is also probably linked with the temporal activation kinetics of IRE1α and PERK. In addition, K145 induces IRE1-dependent ‘alarm’ activation of JNK and p38 MAPK. The molecular basis of UPR activation by SK2 inhibitors involves elevation of ceramide and changes in lipid composition of the ER membrane have been shown to directly activate IRE1α and PERK independently of lumenal unfolded proteins [5]. Importantly, Wallington-Beddoe et al. [2] demonstrate that the findings in MM cell lines are recapitulated *in vivo* using the 5TGM1 C57BL/KaLwRij murine model of myeloma.

These findings raise a number of important issues. First, many of the effects observed with the SK2 inhibitor, K145, on ER stress/UPR and combination with bortezomib are recapitulated with the SK2 inhibitor, ABC294640 [2]. However, a previous study reported that ABC294640 directs c-Myc and Mcl-1 for proteasomal degradation and increases pro-apoptotic Noxa gene transcription to suppress growth of MM [1]. This might represent an early event in ER stress via deployment of ER-associated degradation (ERAD) that is then switched to CHOP/caspase-3-induced apoptosis in the presence of bortezomib. Second, ABC294640 also inhibits dihydroceramide desaturase (Des1) [6, 7] that we have suggested might be a ‘gate-keeper’ of the proteasome [6]. Third, we have also shown that the dual SK1/SK2 and Des1 inhibitor, SKi (4-([4-(4-chlorophenyl)thiazol-2-yl] amino) phenol) induces an ER stress/UPR in T-cell lymphoblastic leukemic (T-ALL) cells that results in a protective cell survival autophagy [8]. Indeed, ER stress/UPR via IRE1α-JNK is linked with Beclin 1 regulation that can orchestrate autophagic responses. In contrast, the SK2 selective inhibitor, (*R*)-FTY720 methylether failed to induce an ER stress/UPR response yet promoted autophagic death of T-ALL cells [8]. Clearly, the role of ER stress/UPR and regulation by SK2 has some cancer specific context, but it would be interesting to evaluate the role of autophagy in the apoptotic process induced by SK2 inhibitors in MM cells

Wallington-Beddoe et al. [2] place ER stress/UPR at the centre of sphingolipid action and specifically ceramide/sphingosine/S1P regulation by SK2 in MM. Perhaps more significantly, they have demonstrated therapeutic potential for combined treatment using SK2 inhibitors and bortezomib. These findings provide impetus for the development of nM potent SK2 inhibitors that can be translated to the clinic to treat myeloma.
